# Greenhouse test of spraying dsRNA to control the western flower thrips, *Frankliniella occidentalis*, infesting hot peppers

**DOI:** 10.1186/s12896-023-00780-y

**Published:** 2023-04-04

**Authors:** Falguni Khan, Minlee Kim, Yonggyun Kim

**Affiliations:** 1 Department of Plant Medicals, College of Life Sciences, Andong National University, Andong, 36729 Korea; 2 Genolution, Inc, Seoul, 05836 Korea

**Keywords:** RNA interference, dsRNA, Insecticide, *Frankliniella occidentalis*, vATPase

## Abstract

**Background:**

The western flower thrips *Frankliniella occidentalis* is an insect pest that damages various crops, including hot peppers. It is a vector of a plant pathogen, tomato spotted wilt virus. To control this pest, chemical insecticides have been used in the past, but the control efficacy is unsatisfactory owing to rapid resistance development by *F. occidentalis*.

**Methodology:**

: This study reports a novel control technology against this insect pest using RNA interference (RNAi) of the vacuolar-type ATPase (*vATPase*) expression. Eight subunit genes (*vATPase-A* ∼ *vATPase-H*) of vATPase were obtained from the *F. occidentalis* genome and confirmed for their expressions at all developmental stages.

**Results:**

Double-stranded RNAs (dsRNAs) specific to the eight subunit genes were fed to larvae and adults, which significantly suppressed the corresponding gene expressions after 24-h feeding treatment. These RNAi treatments resulted in significant mortalities, in which the dsRNA treatments at ∼2,000 ppm specific to *vATPase-A* or *vATPase-B* allowed complete control efficacy near 100% mortality in 7 days after treatment. To prevent dsRNA degradation by the digestive proteases during oral feeding, dsRNAs were formulated in a liposome and led to an enhanced mortality of the larvae and adults of *F. occidentalis*. The dsRNAs were then sprayed at 2,000 ppm on *F. occidentalis* infesting hot peppers in a greenhouse, which resulted in 53.5–55.9% control efficacy in 7 days after treatment. Even though the vATPases are conserved in different organisms, the dsRNA treatment was relatively safe for non-target insects owing to the presence of mismatch sequences compared to the dsRNA region of *F. occidentalis*.

**Conclusion:**

These results demonstrate the practical feasibility of spraying dsRNA to control *F. occidentalis* infesting crops.

**Supplementary Information:**

The online version contains supplementary material available at 10.1186/s12896-023-00780-y.

## Background

The western flower thrips, *Frankliniella occidentalis*, is classified as Thripidae among the different families of the Tenebrantia suborder. It has a wide host spectrum and causes massive economic damage to various ornamental and other horticultural crops [1]. In particular, it causes both direct and indirect damage to crops by feeding or ovipositing on the host plants and by transmitting a plant pathogen called tomato spotted wilt virus (TSWV) [2]. The high potential of this insect to invade and colonize new habitats has been well documented in many countries [3]. Indeed, it was found in most of the counties in Korea within a decade since its first detection in 1993 [4].

Relatively small body size, cryptic behavior, short generation time, and high reproduction rate allow the thrips to frequently attack crops [5]. Chemical insecticides have been used to control the thrips but are not always satisfactory in their control efficacies because of rapid development of insecticide resistance [3]. Usually, plants develop a series of constitutive and inducible defense mechanisms against insect-feeding stress. Breeding insect-resistant varieties has been considered effective for controlling thrips. However, insects also evolve antidefense mechanisms, including behavioral, physiological, and biochemical adaptations, to increase survival and reproduction against the host plants [6]. Indeed, *F. occidentalis* induced detoxifying potential by elevating its glutathione-S-transferase activity against secondary metabolites, such as tannins, alkaloids, total phenols, flavonoids, and lignin [7].

RNA interference (RNAi) is defined as sequence-specific silencing of a target gene expression by shortening the mRNA lifetime [8]. In the RNAi pathway, the target mRNA is degraded by hybridization with its specific antisense RNA derived from a double-stranded RNA (dsRNA) after a catalytic activity of an RNase (= Dicer) [9]. RNAi has provided a novel and powerful reverse genetic tool for identifying gene functions and shows great potential in pest management [10]. By targeting genes essential for the survival of specific pest insects, RNAi can be used to selectively kill such pest insects without adversely affecting the non-target species [11].

The present study was aimed at developing novel control tactics against *F. occidentalis* using RNAi against the vacuolar-type ATPase (vATPase) gene. vATPase is highly conserved among eukaryotic organisms via its catalytic activity to acidify various intracellular compartments by pumping protons across the plasma membranes with the help of ATP hydrolysis [12]. It has two domains, namely a membrane V0 domain performing H^+^ channel hydrolysis and an external V1 domain performing ATP hydrolysis [13]. The V1 domain is composed of eight subunits (A-H), in which three copies of the A and B subunits are catalytic [14]. Baum et al. [15] demonstrated the control efficacy of dsRNA specific to vATPase-A against a corn rootworm. In addition, various other insect pests, such as the fruit fly (*Drosophila melanogaster*), beetle (*Tribolium castaneum*), aphid (*Acyrthosiphon pisum*), and moth (*Manduca sexta*), were selectively exterminated when fed species-specific dsRNA-targeting vATPase transcripts [11]. This motivated us to re-evaluate the control efficacy of dsRNA specific to vATPase gene of *F. occidentalis*.

## Methods

### Insect rearing

The larvae and adults of *F. occidentalis* were collected from the National Academy of Agricultural Sciences (Jeonju, Korea). The rearing conditions were maintained at a temperature of 25 ± 2 °C, with photoperiod of 14:10 h (L:D) and 65 ± 5% relative humidity. A circular breeding container (100 ⋅ 40 mm, SPL, Seoul, Korea) was used to rear the thrips via continuous supply of soybean, *Phaseolus coccineus*, as a diet. The beans were germinated, and the sprouted seed kernels were provided as a diet for the larvae and adults.

### Bioinformatics

Eight subunit sequences of the *F. occidentalis* vATPase V1 domain (A-H) were obtained with GenBank accession numbers of XM_026418961.1, KP234253.1, XM_026421419.1, XM_026424871.1, XM_026438253.1, XM_026421632.1, XM_026416289.1, and XM_026438737.1. The resulting sequences were subjected to open reading frame (ORF) analysis using ORFfinder (https://www.ncbi.nlm.nih.gov/orffinder/).

### RNA extraction, cDNA synthesis, RT-PCR, and RT-qPCR

RNA samples were extracted from all developmental stages with around 50 individuals per stage using Trizol reagent (Invitrogen, Carlsbad, CA, USA) under manufacturer’s instructions. The RNA concentrations were measured using a spectrophotometer (NanoDrop, Thermo Scientific, Wilmington, DE, USA) after resuspending in nuclease-free water. The extracted RNAs (60 ng per reaction) were used for cDNA synthesis using RT-Premix (Intron Biotechnology, Seoul, Korea) containing the oligo dT primer based on manufacturer’s instructions.

The synthesized cDNAs were used for PCR amplification using DNA Taq polymerase (GeneALL, Seoul, Korea) with gene-specific forward and reverse primers under the following conditions: 94 °C for 5 min for the initial heat treatment, followed by 35 cycles of denaturation at 94 °C for 1 min, different annealing temperatures (52–55 °C) for 1 min, and extension at 72 °C for 1 min. The PCR was terminated with a final chain extension at 72 °C for 10 min. Each PCR mixture (25 µL) consisted of the DNA template, dNTP (2.5 mM each), 10 pmol of each forward and reverse primer, and Taq polymerase (2.5 unit/µL).

Quantitative PCR (qPCR) was conducted using a Real-time PCR machine (Step One Plus Real-Time PCR System, Applied Biosystems, Singapore) and the Power SYBR Green PCR Master Mix (Life Technologies, Carlsbad, CA, USA) according to the procedures of Bustin et al. [16]. The reaction mixture (20 µL) contained 10 µL of the Power SYBR Green PCR Mix, 2 µL of the cDNA template (60 ng/µL), and 1 µL each of the forward and reverse primers. An elongation factor (*EF1*) was used as the reference gene. The quality of the PCR products was assessed by melting curve analysis. Quantitative analysis was performed using the comparative CT (2^−∆∆CT^) method [17], where each experiment was replicated three times with individually prepared samples.

### dsRNA preparation

Template DNAs were amplified with gene-specific primers containing T7 promoter sequence at 5’ end. These were used for in vitro transcription using the MEGAscript RNAi Kit (Ambion, Austin, TX, USA) according to manufacturer’s instructions. The resulting dsRNAs were mixed with Metafectene PRO (Biontex, Plannegg, Germany), a transfection reagent, at 1:1 ratio and incubated at room temperature for 30 min to form liposomes. For field assays, two dsRNAs specific to *vATPase-A* and *vATPase-B* were produced at a large scale by Genolution Inc. (Seoul, Korea). In the production, three grades of dsRNA quality were produced: ‘Grade 1’ represented the dsRNA mixture without any purification, ‘Grade 2’ was a dsRNA product purified by alcohol precipitation, and ‘Grade 3’ was a dsRNA product purified by 0.22 μm membrane filtration.

### Insecticidal efficacy test of dsRNA against *F. occidentalis* in the laboratory

Before laboratory bioassays, RNAi efficacy was assessed. Different dsRNAs (dsATPaseA∼dsATPaseH) specific to the eight *vATPase* subunits were prepared using the MEGAscript kit described above. These dsRNAs were fed to the larvae and adults through the bean diet. The beans were soaked in the dsRNA suspension for 20 min. After removing the excess moisture, the treated beans were supplied to 10 individuals of *F. occidentalis* in a circular breeding container (100 ⋅ 40 mm) (= an experimental unit). After 24 h of dsRNA-treated diet feeding, untreated fresh beans were supplied to the test thrips. After the 24-h dsRNA treatment, the RNAi efficiencies were measured over 0–24 h by RT-qPCR. Each treatment was replicated three times. As a control, the viral gene *CpBV302* [18] was used.

For bioassay after the 24-h of dsRNA-treated diet feeding, untreated fresh beans were supplied to the test thrips. Mortality was monitored for 7 days after treatment (DAT). Each treatment used 10 individuals and was replicated three times.

### Control efficacy of dsRNA against *F. occidentalis* in the greenhouse

The study greenhouse contained five rows of cultivated young (about 30 cm height) hot peppers, where the average distance between adjacent rows was around 50 cm, and the average distance between adjacent plants was 40 cm. Each plant was considered as one experimental unit and infested with 80–90 thrips before application of the dsRNA mixture. These experimental units were deployed under a randomized complete block design. Each treatment was replicated with three blocks. To cover the entire hot pepper plant including both sides of leaves, each spray volume per plant was ~ 6.5 mL, which contained 1,994.2 µg/mL for dsATPaseA or 2,025.1 µg/mL for dsATPaseB. For the control, the same volume of water was sprayed.

### Influences of dsATPaseA and dsATPaseB on non-target insects

The non-target insects included *Thrips tabaci*, *Plutella xylostella*, *Tenebrio molitor*, and *Tribolium castanum*. These test insects were obtained from laboratory cultures of *T. tabaci* [19], *P. xylostella* [18], *T. molitor* [20], and *T. castaneum* [21]. The larval stages of these species were used for the bioassays. dsATPase (2,000 ppm) was fed to the larvae with the treated diets via welsh onion for *T. tabaci*, cabbage for *P. xylostella* and *T. molitor*, and nuts for *T. castaneum*. Each treatment used 10 individuals and was replicated three times. The mortality was assessed at 7 DAT under the rearing conditions.

### Statistical analysis

All studies were analyzed with one-way ANOVA using PROC GLM of SAS program [22]. The mortality data were subjected to arcsine transformation and used for ANOVA. The means were compared with the least-squares difference (LSD) test. The study experiments involved three biologically independent replicates that were plotted as mean ± standard error using Sigma plot. The BioRender program was used for the schematic diagram (https://biorender.com/).

## Results

### Expression profiles of eight subunit genes of *vATPase* in *F. occidentalis*

**Fig. 1:**
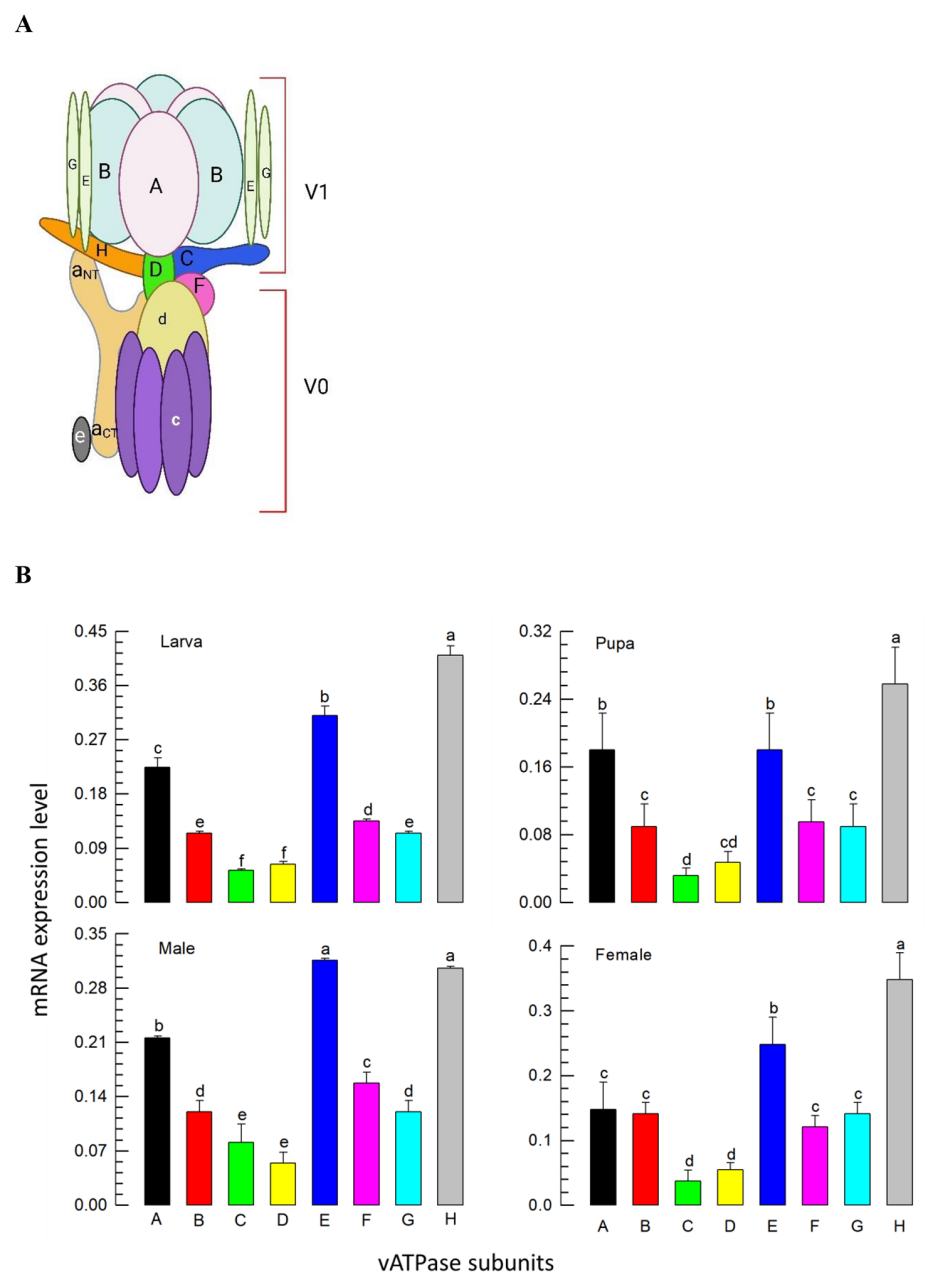
Identification and expression profiles of *vATPase* subunits of *F*. *occidentalis*. **A** Molecular structures of the vATPase domains (subunits): V_0_ (a-e) and V_1_ (A-H). Redrawn from Chen et al. [23]. **B** Expression analysis of *vATPase* subunits at different developmental stages. An elongation factor *EF1* was used to normalize the expression levels at different stages. Each measurement was replicated with three independent samplings. The letters above the standard deviation bars indicate significant differences among the means at Type I error = 0.05 (LSD test)

Eight subunits (‘A-H’) of vATPase V1 domain (Fig. 1A) were obtained from *F. occidentalis* genome. These genes were highly similar (73–99%) to other insect orthologs in the predicted amino acid sequences (Table S2). All eight genes were expressed in the larvae, pupae, and adults (Fig. 1B). However, the expression levels of the different subunits varied at each stage. Moreover, similar expression patterns were exhibited among the different stages, with relatively high expressions of *vATPase-E* and *vATPase-H*.

### Individual RNAi of the eight *vATPase* subunits and subsequent insecticidal activities

**Fig. 2:**
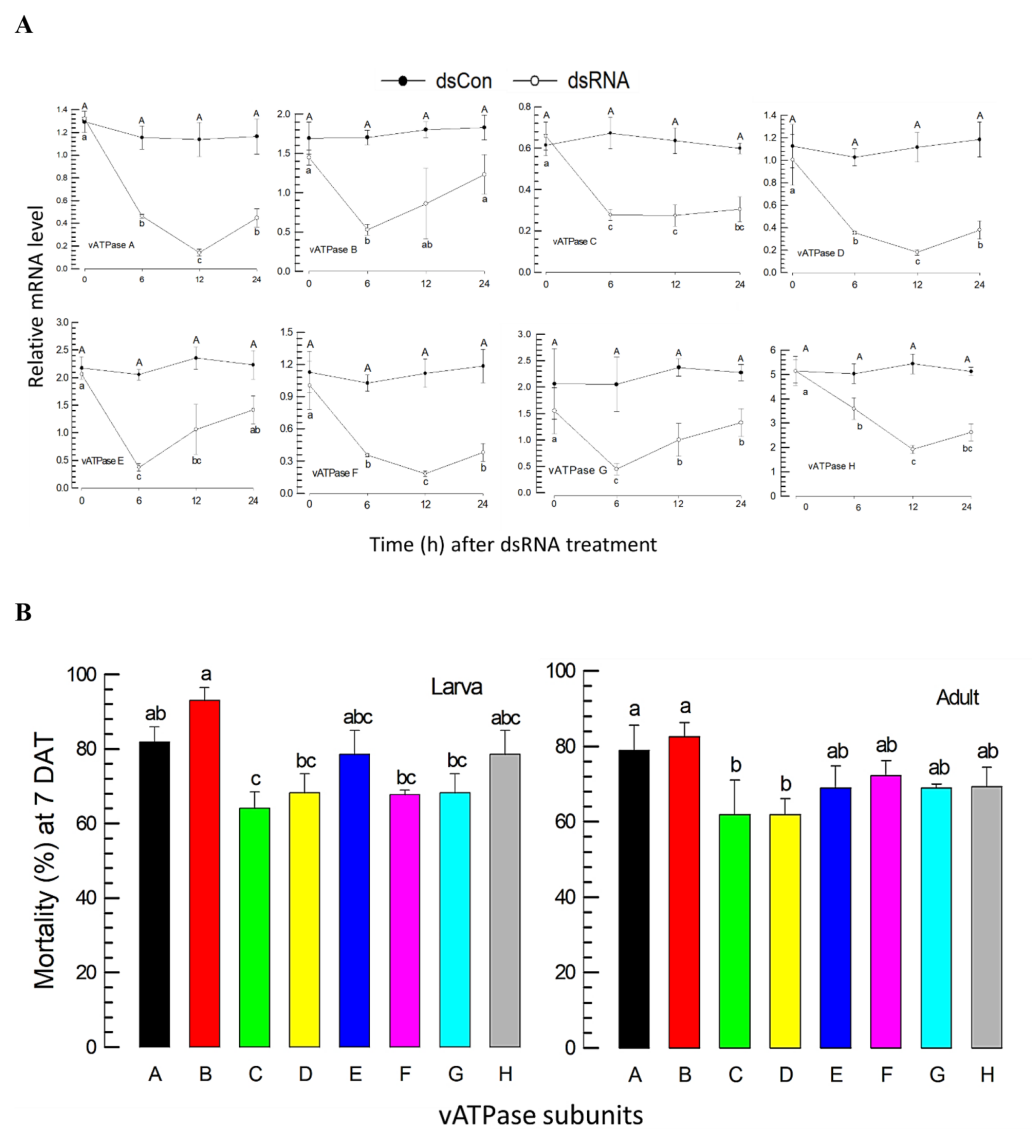
RNAi and bioassays by feeding dsRNA (500 ppm) specific to *vATPase* subunit (A-H) genes against *F*. *occidentalis*. **A** RNAi efficiencies of eight individual dsRNAs in adults. A viral gene *CpBV302* was used as the control dsRNA (dsCON). An elongation factor *EF1* was used to normalize the expression levels. Each measurement was replicated with three independent samplings. **B** Bioassays of the individual RNAi against larvae and adults. Mortality was assessed at 7 days after treatment (DAT) and corrected by the mortality of control treatment obtained by feeding dsCON. Each treatment used 10 individuals and was replicated three times. The letters above the standard deviation bars indicate significant differences among the means at Type I error = 0.05 (LSD test)

RNAi specific to each of the eight *vATPase* subunits was performed by feeding a diet soaked in dsRNA suspension at 500 ppm (Fig. 2). After 24-h of feeding dsRNA, the expression level of each subunit was monitored over 24 h by RT-qPCR (Fig. 2A). All RNAi treatments reduced the expression levels of the target *vATPase* subunits by more than 50%. Except for *vATPase-G*, the other *vATPase* subunits exhibited the highest reductions in expression levels 12 h after dsRNA feeding. Under these RNAi conditions, the treated thrips suffered from significant (*P* < 0.05) mortalities. In both the larval and adult stages, more than 80% mortalities were recorded for treatments with dsRNA specific to *vATPase-A* or *vATPase-B*, which were used for the subsequent bioassays (Fig. 2B).

### Production of effective dsRNAs at a large scale and their control efficacies against *F. occidentalis*

**Fig. 3:**
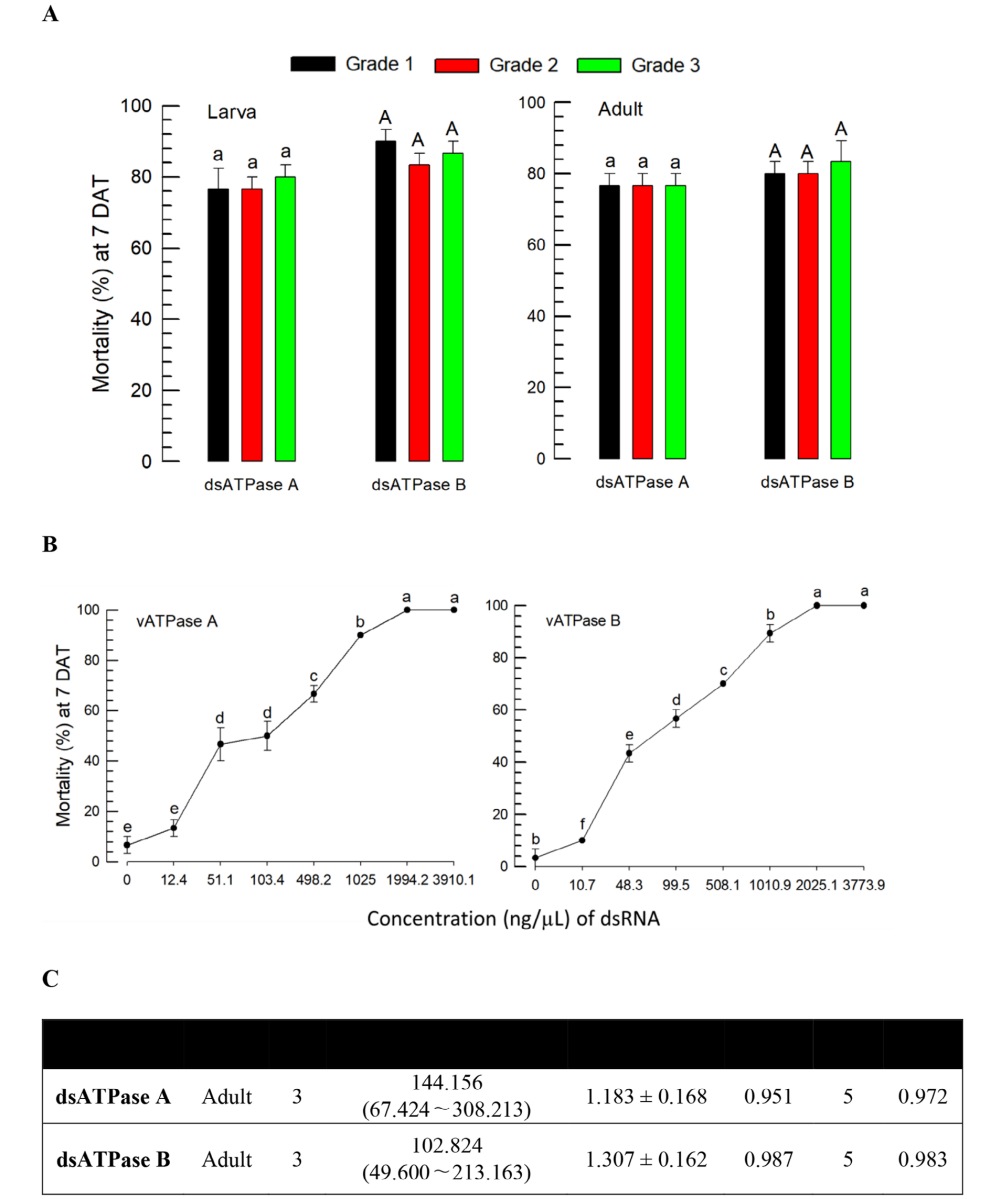
Evaluation of the company-made dsRNAs for insecticidal activities against *F. occidentalis*. Two dsRNAs specific to *vATPase-A* and *vATPase-B* were produced at a large scale. **A** Comparative analysis of the insecticidal activities of three different grades of dsRNAs based on purity. A viral gene *CpBV302* was used as the control dsRNA. Mortality was assessed at 7 days after treatment (DAT) and corrected by the mortality of control treatment obtained by feeding the control dsRNA. **B** Dose-mortality curves of the two Grade-1 dsRNAs against adult thrips. Test dsRNA suspensions were applied to the thrips by the feeding method. Each treatment used 10 individuals and was replicated three times. The letters above the standard deviation bars indicate significant differences among the means at Type I error = 0.05 (LSD test) for each developmental stage or target gene. **C** Median lethal concentrations (LC_50_) of the two dsRNAs against adult thrips

For the field assays, the two most effective dsRNAs specific to *vATPase-A* and *vATPase-B* were produced at a large scale in three grades, which differed in purity levels from Grade 1 to Grade 3 (Fig. 3A). All three grades were prepared at 500 ppm of active gradient and applied to the thrips. These dsRNAs also showed high control efficacies against both larvae and adults, which were observed in treatments using dsRNAs prepared with the MEGAscript kit. However, the control efficacies were not different among the different purity levels of the dsRNAs produced at a large scale.

For the Grade-1 dsRNAs produced at a large scale, the dose-mortality was assessed against the adults of *F. occidentalis* (Fig. 3B). With increase in the dsRNA amount, the thrips suffered high mortalities. At around 2,000 ppm, both dsRNAs resulted in 100% mortality. With respect to the median lethal dose (LC_50_), however, the dsRNA specific to *vATPase-B* was more potent than that specific to *vATPase-A* (Fig. 3C).

### Comparative toxicity analyses of potent dsRNAs against *F. occidentalis*

**Fig. 4:**
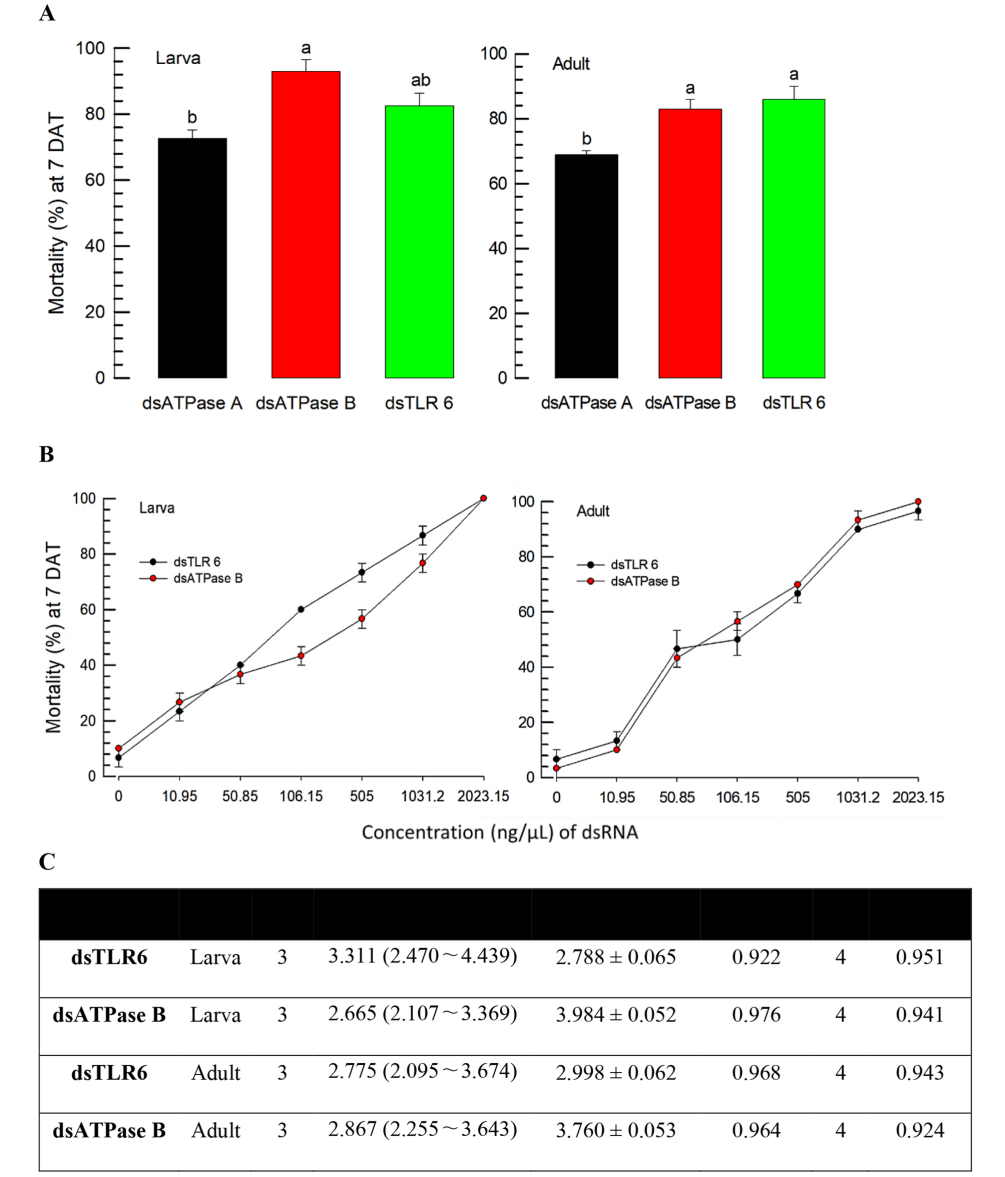
Insecticidal activities among the three dsRNAs (dsATPase A, dsATPase B, and dsTLR6) specific to *vATPase-A*, *vATPase-B*, and *TLR6*, respectively, against *F. occidentalis*. **A** Comparative analysis of the dsRNAs by feeding a dose of 500 ppm to both larvae and adults. A viral gene *CpBV302* was used as the control dsRNA. Mortality was assessed at 7 days after treatment (DAT) and corrected by the mortality of control treatment obtained by feeding the control dsRNA. **B** Dose-mortality curves of the two dsRNAs against the larvae and adults. Test insecticide suspensions were applied to the thrips by the feeding method. Each treatment used 10 individuals and was replicated three times. The letters above the standard deviation bars indicate significant differences among the means at Type I error = 0.05 (LSD test). **C** Median lethal time (LT_50_) of the two dsRNAs against larvae and adults

A previous screening [24] among 57 gene-specific dsRNAs proposed *TLR6* as a potent target to effectively exterminate *F. occidentalis*. The insecticidal activity of the dsRNA specific to TLR6 was compared with those of dsRNAs specific to *vATPase-A* and *vATPase-B* by the feeding bioassay used in our current study at 500 ppm of dsRNA concentration (Fig. 4A). In both larvae and adults, the dsRNA specific to *vATPase-B* or *TLR6* was more potent than the treatment with the dsRNA specific to *vATPase-A*. The two potent dsRNAs were further assessed at different doses (Fig. 4B). There were no significant differences for different doses on the larvae (*F* = 5.82; df = 1, 28; *P* = 0.0524) and adults (*F* = 0.08; df = 1, 6; *P* = 0.7868). In addition, these two dsRNAs were not very different at the median lethal doses (Fig. 4C).

### Enhanced insecticidal activity by dsRNA formulation with liposome against *F. occidentalis*

**Fig. 5:**
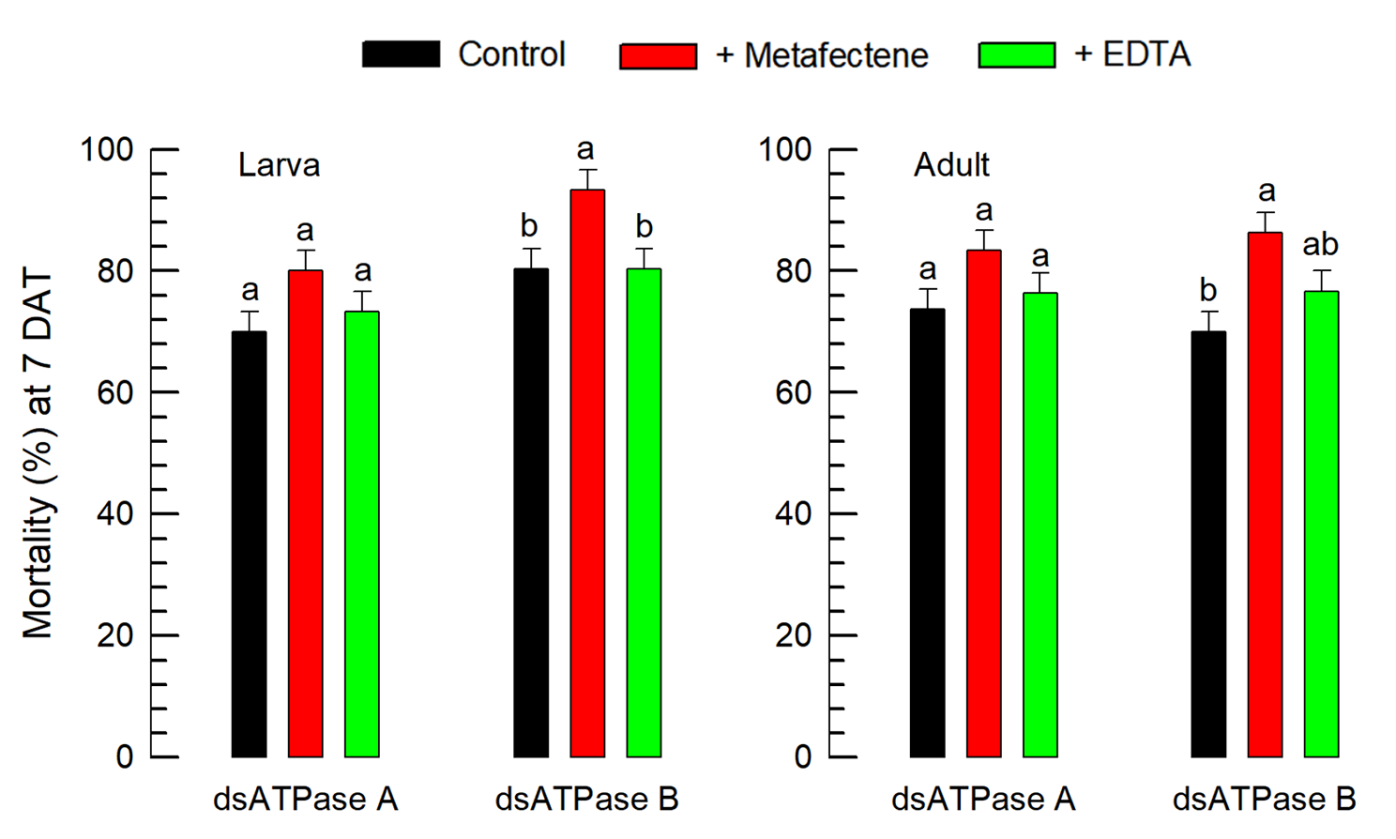
Enhancement of insecticidal activity by liposome formation of dsRNA against *F. occidentalis*. The **‘**Control’ treatment represents plain dsRNA without any formulation. Liposome formation (‘+ Metafectene’) used a lipofectin reagent, metafectene, in a 1:1 volume ratio mixture with the dsRNA suspension (Grade 1). ‘+EDTA’ represents the addition of 3% EDTA to the dsRNA suspension. All dsRNA treatments used a feeding assay at the dose of 500 ppm. A viral gene *CpBV302* was used as the control dsRNA. Mortality was assessed at 7 days after treatment (DAT) and corrected by the mortality of control treatment obtained by feeding the control dsRNA. Each treatment used 10 individuals and was replicated three times. The letters above the standard deviation bars indicate significant differences among the means at Type I error = 0.05 (LSD test)

To prevent degradation of the dsRNA during feeding, EDTA was added to inactivate the degradation enzyme(s) or liposome formulation was devised (Fig. 5). The addition of EDTA to the dsRNAs specific to *vATPase-A* and *vATPase-B* did not significantly increase the control efficacy against larvae or adults. However, the liposome formulation using metafectene significantly enhanced the insecticidal activity of the dsRNA specific to *vATPase-B* against both developmental stages of *F. occidentalis*. Enhanced control efficacy of liposome formation was detected in the treatment with the dsRNA specific to *vATPase-A*, but not significantly different compared to the control dsRNA treatment without any addition.

### Field assays of *vATPase-A* and *vATPase-B* against *F. occidentalis* infesting hot pepper

**Fig. 6:**
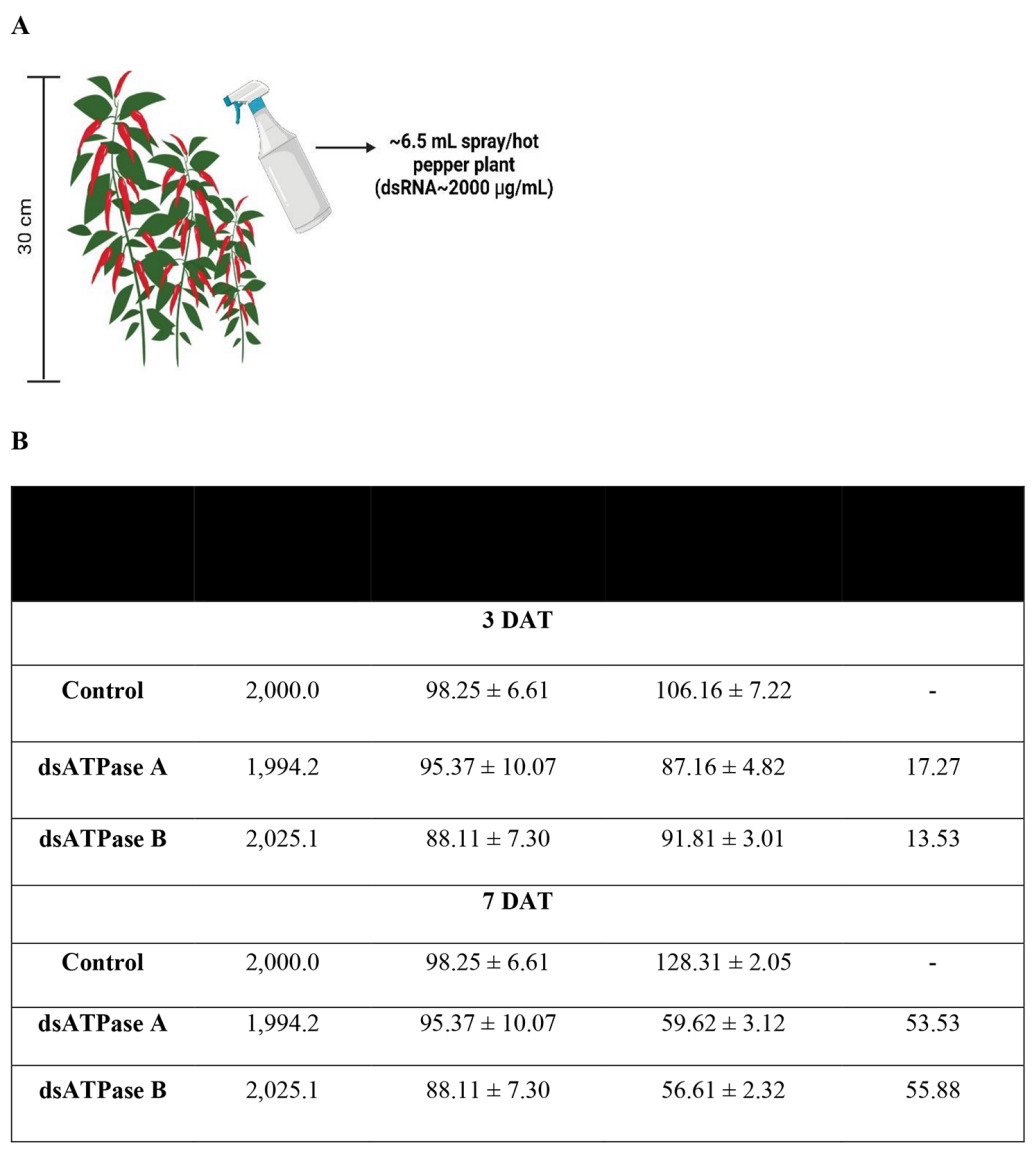
Field test of dsRNAs specific to vATPase for controlling *F. occidentalis* infesting hot peppers in a greenhouse. **A** Schematic diagram of spraying dsRNAs in the greenhouse, where the row-to-row distance was around 50 cm, and the plant-to-plant distance was 40 cm. Each treatment was replicated under a randomized complete block design, in which each plant represented an experimental unit. Control treatment represents water spraying without any dsRNAs. **B** Control efficacies of two dsRNAs specific to *vATPase-A* and *vATPase-B*. Mortalities were assessed at 3 and 7 days after treatment (DAT).

Liposome formulation of dsRNA (∼2,000 ppm) specific to *vATPase-A* or *vATPase-B* was sprayed on *F. occidentalis* infesting hot peppers (Fig. 6). The relatively young host plants were evenly sprayed with ∼6.5 mL of the dsRNA suspension (Fig. 6A). Before spraying, each plant had 88–98 thrips, including both larvae and adults (Fig. 6B). Control efficacies were observed at 3 DAT: dsRNA spray targeting *vATPase-A* showed 17.27% control efficacy while that with *vATPase-B* showed 13.53% control efficacy. The control efficacies of the dsRNA treatments increased at 7 DAT, recording 53.53% and 55.88% for dsRNAs specific to *vATPase-A* and *vATPase-B*, respectively.

### Target-specific control efficacy of dsRNA specific to *vATPase-B*

**Fig. 7:**
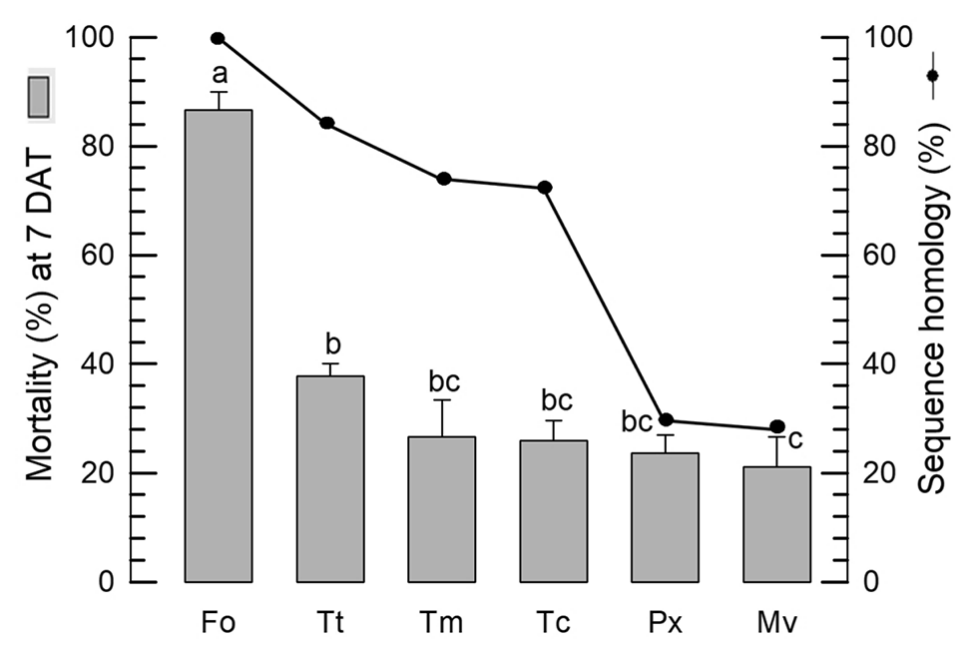
Influence of dsRNA specific to *vATPase-B* against *F*. *occidentalis* (Fo) and non-target insects, namely *Thrips tabaci* (Tt), *Plutella xylostella* (Px), *Maruca vitrata* (Mv), *Tenebrio molitor* (Tm), and *Tribolium castanum* (Tc). Test dsRNAs at 2,000 ppm were applied to the test insects by the feeding method. Mortality was assessed at 7 days after treatment (DAT). Each treatment used 10 individuals and was replicated three times. The letters above the standard deviation bars indicate significant differences among the means at Type I error = 0.05 (LSD test). A viral gene *CpBV302* was used as the control dsRNA. Mortality was assessed at 7 days after treatment (DAT) and corrected by the mortality of control treatment obtained by feeding the control dsRNA. DNA sequences corresponding to the dsRNAs of *F. occidentalis* were compared among these insects (Fig. S2)

Considering the importance and conserved nature of vATPase in all organisms, any non-target damage owing to dsRNA application must be assessed. For this assay, five other insect species were compared with *F. occidentalis* for control efficacies after treatment with dsRNA specific to *vATPase-B* (Fig. 7). As expected, > 80% control efficacy of the dsRNA treatment was detected against *F. occidentalis* while the dsRNA treatment showed less than 40% control efficacies for the other non-target insects. When the DNA sequences corresponding to dsRNA were compared among these insects, the sequence identities appeared to be positively correlated (*r* = 0.6582) with the control efficacies, although the correlation coefficient was not significant (*P* = 0.1552). Interestingly, the identical 20-nucleotide (∼ short interfering RNA (siRNA) size after dsRNA process) site was detected between vATPase genes of *F. occidentalis* and *T. tabaci* (Fig. S2). *T. tabaci* was highly influenced by dsRNA specific to *vATPase-B* of *F. occidentalis* among the selected non-target species.

## Discussion

This study tested the insecticidal activities of dsRNAs specific to *vATPase* against *F. occidentalis*. In the RNAi application, dsRNAs were delivered by oral administration via a diet coated with the dsRNA solutions. The typical dsRNA delivery methods include microinjection, feeding, and soaking [25]. Among these delivery methods, the current study used feeding of dsRNA against *F. occidentalis* and showed effective RNAi efficacy resulting in significant insecticidal activities. Relatively high RNAi efficiencies via feeding dsRNA are usually observed in *F. occidentalis* unlike other sucking insects such as aphids because of the unique feeding behavior of the thrips exhibiting a pierce-sucking type followed by feeding the exuding plant juice from the broken plant tissues probably containing the treated dsRNA on the plant surface [24]. In addition, Whitten et al. [26] successfully fed *F. occidentalis* media containing dsRNA-expressing bacteria, which resulted in significant mortality of the insects, especially those at the larval stage, by targeting the alpha-tubulin gene. RNAi has been used to control gene expression during the development of eukaryotes, including insects [27]. In response to dsRNA, cellular enzymes Dicer or Dicer-like proteins are activated to cleave it into double-stranded siRNAs (20–24 nucleotides). One of the siRNA strands is loaded onto the RNA-induced silencing complex (RISC) to surveil the complementary sequences, which then undergo translational arrest or are degraded by the action of the argonaute (AGO) core protein in the RISC complex [28, 29]. In insects, RNAi has been considered for pest control by oral administration of dsRNA [10]. The uptake of dsRNA from the insect gut is mediated either by specific transmembrane proteins or by endocytosis [30]. These suggest the practical application of dsRNA for con-trolling *F. occidentalis* under field conditions. The present study tested the control efficacies of dsRNAs targeting the *vATPase* of *F. occidentalis*.

A genome of *F. occidentalis* encodes all eight subunits of the V1 domain. Individual dsRNAs specific to each of these subunits were tested to compare their insecticidal activities, which showed that dsRNAs specific to *vATPase-A* and *vATPase-B* were relatively the most potent. Three copies of these two subunits form a catalytic V1 domain of the enzyme [14]. This vacuolar-type ATP synthase is involved in solute transport across the plasma membranes as well as lumen pH regulation [31]. Thus, the RNAi of the gene transcripts must have been detrimental to the thrips’ survival. Han et al. [24] screened the RNAi efficiencies of 57 genes of *F. occidentalis*, including *vATPase*, and proposed four dsRNAs specific to the Toll-like receptor 6 (*TLR6*), apolipophorin (*apoLp*), coatomer protein subunit epsilon (*COPE*), and sorting/assembly machinery component (*SAM*) genes to be effective for exterminating thrips by oral administration. However, dsRNAs specific to *vATPase* subunits were highly potent in several insects, including *F. occidentalis*. Indeed, a comparative analysis in the current study showed that the dsRNA specific to *vATPase-B* was equivalent to that of TLR6 in control efficacy against both larvae and adults of *F. occidentalis*. Badillo-Vargas et al. [32] reported a significant decrease in survival and fertility by introducing dsRNA into *F. occidentalis* via microinjection targeting *vATPase-B*. The oral administration of dsRNA specific to *vATPase* was effective by direct feeding in solution rather than feeding via diet in *F. occidentalis* [33]. vATPase is well known for mediating saliva secretion in insects. In the blowfly *Calliphora vicina*, serotonin induces saliva secretion by elevating cAMP levels, which activate protein kinase A to assemble and activate vATPase in the apical membrane [34]. Saliva plays a crucial role in the feeding of *F. occidentalis* because it contains various functional factors for digestion and detoxification against toxic secondary metabolites from the host plants [35]. Thus, its suppression by RNAi would lead to significant lethality against *F. occidentalis*.

Liposome formulation of dsRNA specific to *vATPase-B* slightly enhanced the control efficacy against *F. occidentalis*. This formulation might be effective to minimize dsRNA degradation in the gut lumen from enzymatic attacks [36]. Specifically, dsRNases are suspected to be potent enzymes for degrading dsRNAs and leading to major obstacles influencing successful RNAi [37]. Since dsRNases were first identified in silkworms, several dsRNases have been reported in different insects [38]. These dsRNases participate in dsRNA degradation and reduce RNAi efficiency because the RNAi treatments against these dsRNase genes effectively protect dsRNAs [39]. Liposome formulation is likely to protect the dsRNA from enzymatic attacks by dsRNases, as demonstrated in *Spodoptera litura* [40]. This explains the enhancement of the control efficacy by liposome formation of the dsRNA specific to *vATPase-B* against *F. occidentalis* in our current study.

Spraying dsRNAs specific to *vATPase-A* and *vATPase-B* resulted in significant control efficacies in *F. occidentalis* infesting hot peppers in the greenhouse. However, the control efficacies under field conditions were much lower (∼55%) than those in the laboratory assays at the same treatment concentrations. The use of sprayable dsRNA-based pest control technology was proven to be effective in the Colorado potato beetle *Leptinotarsa decemlineata* [41]. However, this dsRNA control technique was applicable owing to the differential sensitivities among the different insects [42]. In particular, the highly susceptible insects appeared to exhibit systemic RNAi, in which the dsRNA was not only capable of entering the gut cells but also spread to other tissues [43]. The relatively efficient RNAi in *F. occidentalis* via feeding delivery suggests that systemic RNAi may operate in this insect species. This needs to be clarified in future studies. In addition, subsequent RNAi studies must assess several other factors that contribute to efficient RNAi, such as core RNAi machinery components and dsRNA metabolism, in *F. occidentalis*. Moreover, the low control efficiency under field conditions suggests environmental factors contributing to RNAi efficiency, which should be protected by development of an optimal formation such as chitosan nanoparticle [44].

The dsRNA specific to *vATPase-B* was relatively safe on non-target insects. The hazard to these non-target organisms appeared to be positively correlated with the sequence similarity among species in the dsRNA region of *vATPase-B*. A recent environmental risk assessment of dsRNAs expressed in a genetically modified maize variety against different non-target organisms, including pollinators and pollen feeders, soil dwelling detritivores, predators and parasitoids, aquatic detritivores, insectivorous birds, and wild mammals, showed that they are not harmful at environmentally relevant concentrations [45]. This supports spraying of dsRNAs to control *F. occidentalis* in agricultural fields.

Altogether, feeding of dsRNA was effective for suppressing the target gene expressions in *F. occidentalis*. The suppression of *vATPase-B* expression by RNAi was lethal to *F. occidentalis*. Thus, RNAi via dsRNA feeding allows a novel strategy against *F. occidentalis* for sustainable agriculture because it allows greater specificity than chemical insecticides [32]. Indeed, a commercial product of the dsRNA via mass production was shown to be toxic to the thrips. This paves the path for practical application of dsRNAs specific to *vATPase-B* to control field populations of *F. occidentalis*.

## Electronic supplementary material

Below is the link to the electronic supplementary material.

Supplementary Material 1

## Data Availability

All data generated or analyzed during this study are included in this published article and its supplementary information.

## Abbreviations

TSWV: Tomato spotted wilt virus
RNAi: RNA interference
dsRNA: double-stranded RNA
vATPase: Vaculolar-type ATPase
DAT: Days after treatment
LSD: Least-squares difference
RISC: RNA-induced silencing complex
AGO: Argonaute
TLR: Toll-like receptor
