# Supplementary material for: Greenhouse test of spraying dsRNA to control the western flower thrips, Frankliniella occidentalis, infesting hot peppers
Source: BMC Biotechnol. 2023 Apr 4;23:10. doi: 10.1186/s12896-023-00780-y (PMC10074877; doi:10.1186/s12896-023-00780-y)
Supplement: Supplementary file 1 — Supplementary Material 1 [file 12896_2023_780_MOESM1_ESM.docx]

**Supplementary data**

**Table S1** Primers used in the present study.

**Table S2** Sequence homologies of eight subunits (*vATPase-A*–*vATPase-H*) of *F. occidentalis* with their orthologs using the Blast search engine of NCBI.

**Fig. S1** Expression analysis of the Toll-like receptor 6 (*TLR6*) at different developmental stages of *F. occidentalis*. An elongation factor, *EF1*, was used to normalize the expression levels at different stages. Three replications were used per treatment. The letters above the standard deviation bars indicate significance differences among the means at Type I error = 0.05 (LSD test).

**Fig. S2** Sequence alignment of *vATPase-B* in the region used for dsRNA construction in different insects: *F. occidentalis* (Fo), *T. tabaci* (Tt), *M*. *vitrata* (Mv), *P. xylostella* (Px), *T. castanum* (Tc), and *T. molitor* (Tm). Red box indicates the region for dsRNA. GenBank accession numbers of *vATPase-B* are XP_026293141.1 for Fo, GFQQ01002394.1 for Tt, XM_038111438.2 for Px, XM_962751.4 for Tc, and GIPG01050449.1 for Tm.

**Table S1** Primers used in the present study

| vATPase subunit | Sequence (5’-3’) | Uses | Annealing temp (^0^C) | Expected size (bp) |
| --- | --- | --- | --- | --- |
| A | TGACGGTATCCAACGTCCAC | RT-PCR  RT-qPCR | 52.0 | 395 |
|  | TTTGACCAGTCAGCAGTGGG |  |  |  |
| T7+ A | TAATACGACTCACTATAGGGAGATGACGGTATCCAACGTCCAC | RNAi | 52.0 | 441 |
|  | TAATACGACTCACTATAGGGAGATTTGACCAGTCAGCAGTGGG |  |  |  |
| B | CTCGCGAACATGTTTTGGCT | RT-PCR  RT-qPCR | 52.0 | 322 |
|  | GAGCCGTTGAAAACACGACC |  |  |  |
| T7+ B | TAATACGACTCACTATAGGGAGACTCGCGAACATGTTTTGGCT | RNAi | 52.0 | 368 |
|  | TAATACGACTCACTATAGGGAGAGAGCCGTTGAAAACACGACC |  |  |  |
| C | GAACTTGGCAGACCTCGTGA | RT-PCR  RT-qPCR | 52.0 | 455 |
|  | AGTTGACAGGCAGGCCATAC |  |  |  |
| T7+ C | TAATACGACTCACTATAGGGAGAGAACTTGGCAGACCTCGTGA | RNAi | 52.0 | 501 |
|  | TAATACGACTCACTATAGGGAGAAGTTGACAGGCAGGCCATAC |  |  |  |
| D | GAAGCCGCTTTCTCTCTTGC | RT-PCR  RT-qPCR | 52.0 | 238 |
|  | TGACAGCTGCCTGGTAGTTC |  |  |  |
| T7+ D | TAATACGACTCACTATAGGGAGAGAAGCCGCTTTCTCTCTTGC | RNAi | 52.0 | 284 |
|  | TAATACGACTCACTATAGGGAGATGACAGCTGCCTGGTAGTTC |  |  |  |
| E | AGTCCAACAGCAACGTCTGA | RT-PCR  RT-qPCR | 55.0 | 176 |
|  | CTCCCAAACGTCTCCTTGCT |  |  |  |
| T7+ E | TAATACGACTCACTATAGGGAGAAGTCCAACAGCAACGTCTGA | RNAi | 55.0 | 222 |
|  | TAATACGACTCACTATAGGGAGACTCCCAAACGTCTCCTTGCT |  |  |  |
| F | ACCGCCAACCCAATTTTATGG | RT-PCR  RT-qPCR | 55.0 | 252 |
|  | GGTTGAACATGCCCTTCGC |  |  |  |
| T7+ F | TAATACGACTCACTATAGGGAGAACCGCCAACCCAATTTTATGG | RNAi | 55.0 | 298 |
|  | TAATACGACTCACTATAGGGAGAGGTTGAACATGCCCTTCGC |  |  |  |
| G | CGTTTGAAGCAGGCAAAGGA | RT-PCR  RT-qPCR | 55.0 | 139 |
|  | TGGTGTCAGCTTCAATGCGA |  |  |  |
| T7+ G | TAATACGACTCACTATAGGGAGACGTTTGAAGCAGGCAAAGGA | RNAi | 55.0 | 185 |
|  | TAATACGACTCACTATAGGGAGATGGTGTCAGCTTCAATGCGA |  |  |  |
| H | GAACGAACGTTTGCAGGCTT | RT-PCR  RT-qPCR | 55.0 | 121 |
|  | CTCCCTCCAGAATTGAGCCG |  |  |  |
| T7+ H | TAATACGACTCACTATAGGGAGAGAACGAACGTTTGCAGGCTT | RNAi | 55.0 | 167 |
|  | TAATACGACTCACTATAGGGAGACTCCCTCCAGAATTGAGCCG |  |  |  |
| Elongation Factor 1 | TCAAGGAACTGCGTCGTGGAT | RT-qPCR | 52.0 | 160 |
|  | ACAGGGGTGTAGCCGTTAGAG |  |  |  |
| TLR 6 | CCGCCTCACTGACATATCTG | RT-PCR  RT-qPCR | 55.0 | 140 |
|  | TGCGGTTCTGGTGCATGTCG |  |  |  |
| T7+ TLR 6 | TAATACGACTCACTATAGGGAGA CCGCCTCACTGACATATCTG | RNAi | 55.0 | 186 |
|  | TAATACGACTCACTATAGGGAGA TGCGGTTCTGGTGCATGTCG |  |  |  |

**Table S2** Sequence homologies of eight subunits (vATPase-A–vATPase-H) of *F. occidentalis* with their orthologs using the Blast search engine of NCBI.

| Species | Genes | *vATPase-A* of *F. occidentalis* | | |
| --- | --- | --- | --- | --- |
|  |  | Homology score | Identity (%) | E-value |
| *Thrips palmi* | V-type proton ATPase subunit-A | 1243 | 98.04 | 0.0 |
| *Nilaparvata lugens* | V-type proton ATPase subunit-A | 1183 | 91.65 | 0.0 |
| *Vespa mandarinia* | V-type proton ATPase subunit-A | 1183 | 92.64 | 0.0 |

| Species | Genes | *vATPase-B* of *F. occidentalis* | | |
| --- | --- | --- | --- | --- |
|  |  | Homology score | Identity (%) | E-value |
| *Thrips palmi* | V-type proton ATPase subunit-B | 1018 | 99.19 | 0.0 |
| *Drosophila biarmipes* | V-type proton ATPase subunit-B | 993 | 96.55 | 0.0 |
| *Trichoplusia ni* | V-type proton ATPase subunit-B | 990 | 95.94 | 0.0 |

| Species | Genes | *vATPase-C* of *F. occidentalis* | | |
| --- | --- | --- | --- | --- |
|  |  | Homology score | Identity (%) | E-value |
| *Thrips palmi* | V-type proton ATPase subunit-C | 759 | 92.50 | 0.0 |
| *Nilaparvata lugens* | V-type proton ATPase subunit-C | 689 | 86.46 | 0.0 |
| *Tribolium madens* | V-type proton ATPase subunit-C | 687 | 85.64 | 0.0 |

| Species | Genes | *vATPase-D* of *F. occidentalis* | | |
| --- | --- | --- | --- | --- |
|  |  | Homology score | Identity (%) | E-value |
| *Homalodisca vitripennis* | V-type proton ATPase subunit-D | 420 | 90.20 | 6e-147 |
| *Nilaparvata lugens* | V-type proton ATPase subunit-D | 410 | 88.26 | 4e-143 |
| *Aphis gossypii* | V-type proton ATPase subunit-D | 409 | 86.94 | 1e-142 |

| Species | Genes | *vATPase-E* of *F. occidentalis* | | |
| --- | --- | --- | --- | --- |
|  |  | Homology score | Identity (%) | E-value |
| *Thrips palmi* | V-type proton ATPase subunit-E | 352 | 88.05 | 1e-120 |
| *Belonocnema kinseyi* | V-type proton ATPase subunit-E | 292 | 76.99 | 5e-97 |
| *Nilaparvata lugens* | V-type proton ATPase subunit-E | 280 | 73.01 | 6e-92 |

| Species | Genes | *vATPase-F* of *F. occidentalis* | | |
| --- | --- | --- | --- | --- |
|  |  | Homology score | Identity (%) | E-value |
| *Thrips palmi* | V-type proton ATPase subunit-F | 243 | 96.67 | 6e-81 |
| *Bemisia tabaci* | V-type proton ATPase subunit-F | 231 | 89.34 | 3e-76 |
| *Apis florea* | V-type proton ATPase subunit-F | 229 | 86.07 | 2e-75 |

| Species | Genes | *vATPase-G* of *F. occidentalis* | | |
| --- | --- | --- | --- | --- |
|  |  | Homology score | Identity (%) | E-value |
| *Thrips palmi* | V-type proton ATPase subunit-G | 154 | 98.26 | 9e-46 |
| *Apis florea* | V-type proton ATPase subunit-G | 124 | 81.74 | 8e-34 |
| *Tribolium madens* | V-type proton ATPase subunit-G | 122 | 80.87 | 4e-33 |

| Species | Genes | *vATPase-H* of *F. occidentalis* | | |
| --- | --- | --- | --- | --- |
|  |  | Homology score | Identity (%) | E-value |
| *Thrips palmi* | V-type proton ATPase subunit-H | 953 | 94.87 | 0.0 |
| *Aphidius gifuensis* | V-type proton ATPase subunit-H | 778 | 77.59 | 0.0 |
| *Coccinella septempunctata* | V-type proton ATPase subunit-H | 774 | 77.15 | 0.0 |


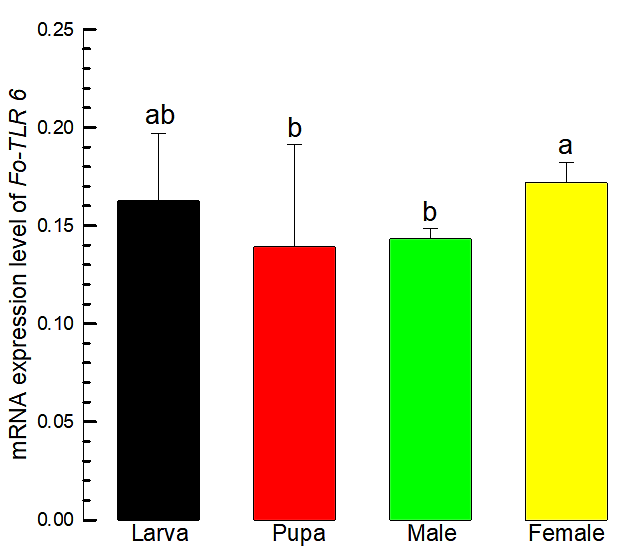


**Fig. S1**


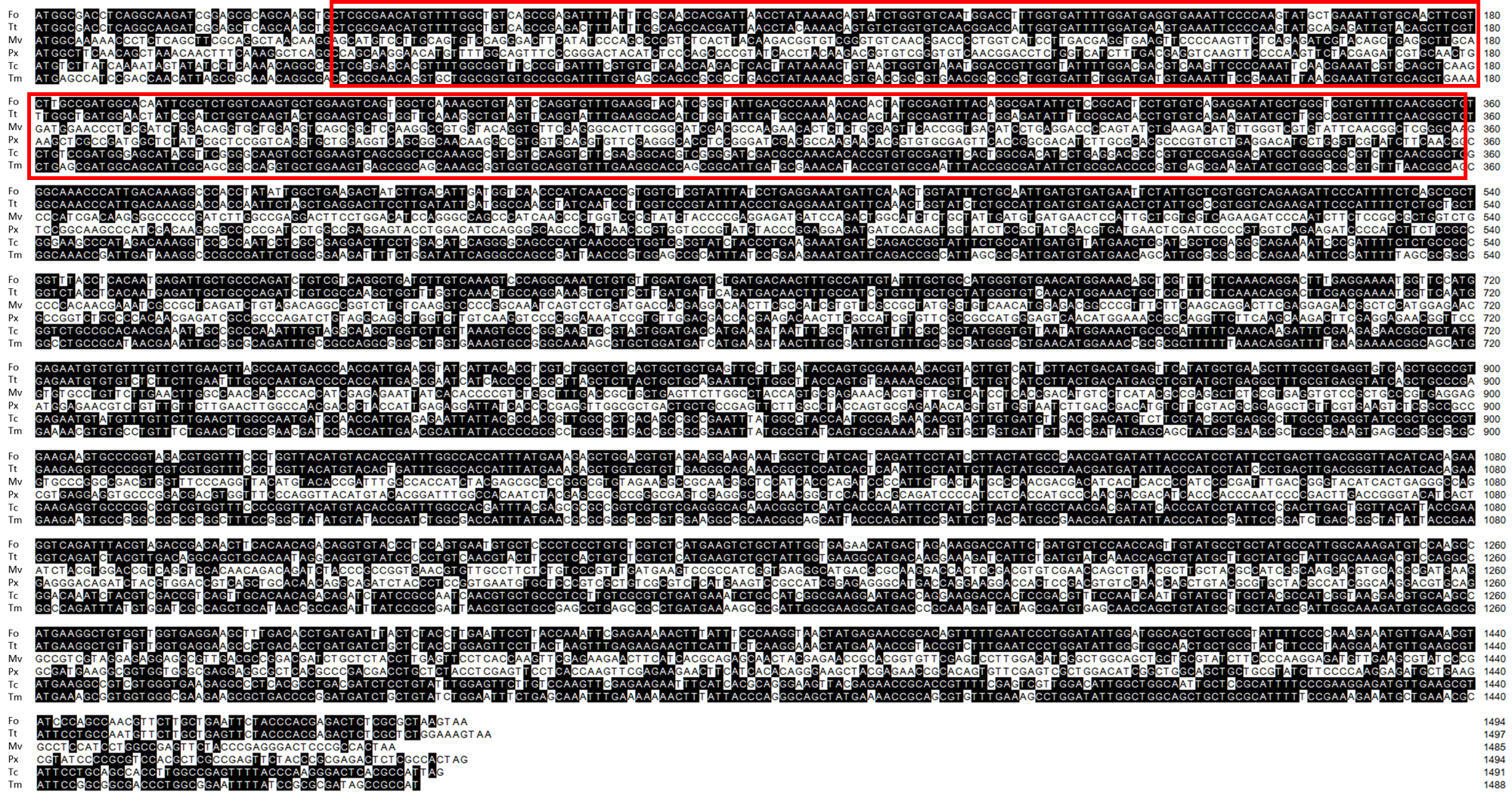


**Fig. S2**
